# Supplementary material for: Patterns of Intron Gain and Loss in Fungi
Source: PLoS Biol. 2004 Nov 30;2(12):e422. doi: 10.1371/journal.pbio.0020422 (PMC532390; doi:10.1371/journal.pbio.0020422)
Supplement: Table S1 — Also available at http://genes.mit.edu/NielsenEtAl/. (4.3 MB ZIP). [file pbio.0020422.st001.zip › NielsenEtAl/html/1291.html]

AN5232.1.NCU04594.1.MG01347.1.FG10039.1


```
 CLUSTAL W (1.82) Multiple Sequence Alignments - Introns Inserted


Sequence 1: MG01347.1	242 aa
Sequence 2: FG10039.1	190 aa
Sequence 3: NCU04594.1	197 aa
Sequence 4: AN5232.1	234 aa
Alignment Length: 256 aa
Number Identitical Residues: 85 aa
Alignment Score (without introns) 4228


MG01347.1 	MPA2FVRFQTAIPIRLTYNPPPSLNHNHSNTTTSSPIRSFHQSPPANMRIPYVPNPPPAS
NCU04594.1	---~-------------------------------------------MRLPYIPNPPPTS
FG10039.1 	---~-------------------------------------------MRIPYVSNPPETK
AN5232.1  	---~-----MNLKSLFRIEARPLLQFRRLSTTRSS-----------KMRLPYAPSEPPTA
          	                  .. .  .    .:: ::           .**:** .. * : 

MG01347.1 	TPEADKAVYERVAARRAPRPLQPLDLALLHSPPVADGWNSFLGAVRTRSSLPADLRELAI
NCU04594.1	S-DAEQAIVDRITARRAPRPLQPLDLTLLHSPPVADGWNSFLGAIRTQTLLPADERELAI
FG10039.1 	T-EEHAAIVKRIEERRAPRPLQSLDLALLHSPHVADGWNSFLGAVRTKTSLSDDLRELAI
AN5232.1  	D-PETAEIYARIAARRRPRPLIPLDLSLLHSPPVADGWNSFLGAIRTQTVVDQGLLELAV
          	       :  *:  ** **** .***:***** ***********:**:: :  .  ***:

MG01347.1 	CRVAVVNTAWYEWMHHAPLAREAGVAES--AMENFVKRTEPLVGSNPSQD-----GLLSD
NCU04594.1	SRVAVCNGAWYEWHHHAPLAVQAGVSEGEDGLGVVKRQEPLLIEEGDQKK-----GGLNE
FG10039.1 	SRVAVCNKAWYEWKHHAPLAVKGGVSEA--GLEAIKQDT---LGERPAE--------LSE
AN5232.1  	SRVAVITQAVYEWNAHAPLALKGGIKPE--ELHAVRTLPSTATGDVDAAVKALGQSALTP
          	.**** . * ***  ***** :.*:      :  .         .     .: ... *. 

MG01347.1 	KQWAVLRYTDEMTRNVRVADETFDLLK-SLFSEREVVEITAT~VACYNCVSRFLVALDV1
NCU04594.1	RQWAAVCLTDEMTRNVKVRDETFERVR-ELFSEREVVELTAI0IACYNCVSRFLVALDV1
FG10039.1 	KQWAVILFTDEMTRNVQVEDETFDRLR-EFFNEQEIVEITAT0VACYNCVSRFLVALDV1
AN5232.1  	QQRAIVRYADEMTQTVKVQDETFAQLQKEGFSDREIVELTTG~IACYNCVSRVLVALDV~
          	:* * :  :****:.*:* ****  ::.. *.::*:**:*:  :********.****** 

MG01347.1 	GEKNG---VGPDD-SAH--
NCU04594.1	GERNG---TGPDD-AAH--
FG10039.1 	GERNG---TDPEAVTGH--
AN5232.1  	GENNAREMKSVDELVAGLK
          	**.*. .  . :   .  .
```
